# Touchscreens for Whom? Working Memory and Age Moderate the Impact of Contingency on Toddlers' Transfer From Video

**DOI:** 10.3389/fpsyg.2021.621372

**Published:** 2021-02-24

**Authors:** Koeun Choi, Heather L. Kirkorian, Tiffany A. Pempek

**Affiliations:** ^1^Department of Human Development and Family Science, Virginia Polytechnic Institute and State University, Blacksburg, VA, United States; ^2^Department of Human Development and Family Studies, University of Wisconsin-Madison, Madison, WI, United States; ^3^Department of Psychology, Hollins University, Roanoke, VA, United States

**Keywords:** touchscreen, contingency, transfer, working memory, toddlers

## Abstract

Toddlers exhibit poor transfer between video and real-world contexts. Contingently responsive video such as that found in touchscreen apps appears to assist transfer for some toddlers but not others. This study investigated the extent to which toddlers' working memory moderates the impact of contingency on toddler's transfer of learning from video. Toddlers (24–36 months; *N* = 134) watched a hiding event on either (a) contingent video that advanced only after touch input or (b) non-contingent video that proceeded automatically. Toddlers then searched for a corresponding object on a felt board. Additionally, toddlers' working memory (WM) was assessed. Findings indicate WM and age moderated the impact of contingency on transfer: Contingency decreased transfer in younger children while increasing transfer among older children. However, this was only true for children with relatively low WM. Contingency had little impact on transfer among children with relatively high WM, regardless of age. Results from this study suggest that WM is one specific moderator that predicts whether toddlers are likely to learn from contingent vs. non-contingent video, yet WM does not operate in isolation. Together, these findings underscore the importance of considering multiple child characteristics when identifying the optimal conditions for toddlers' learning from symbolic media.

## Introduction

Interactive digital media, such as touchscreen applications, have become prevalent in young children's environments (Shuler et al., 2012; Kabali et al., 2015; Rideout, 2017). While infants and toddlers are unlikely to transfer information from non-interactive video to real-world problems (DeLoache, 1987; Barr, 2010; Troseth, 2010), there is emerging, but mixed, evidence that toddlers are more likely to learn from video that is interactive in some way such as video chats (Troseth et al., 2006, 2018; Nielsen et al., 2008; Roseberry et al., 2014; Myers et al., 2017, 2018; Strouse et al., 2018), computer games (Lauricella et al., 2010), and touchscreens (Moser et al., 2015; Choi and Kirkorian, 2016; Kirkorian et al., 2016a; Russo-Johnson et al., 2017). While prior research has examined age-related differences in the impact of interactive and non-interactive media on learning, less attention has been paid to specific cognitive skills that may underlie such age-related differences. Researchers have proposed that working memory (WM) plays a role in children's symbol-mediated learning (Barr and Hayne, 1999; Barr, 2010; Barr et al., 2016; Choi et al., 2018; Hartstein and Berthier, 2018). However, the extent to which WM moderates learning from video that incorporates one type of interactivity, tap-to-play contingency, has not been tested. Thus, the purpose of this study was to examine the moderating role of WM in toddlers' learning from contingent and non-contingent video on touchscreens.

### Working Memory and Symbol-Mediated Learning

WM describes the ability to maintain and update representations in mind for future actions (Baddeley, 1986; Reznick, 2008). WM emerges before 2 years of age (Diamond et al., 1997), rapidly improves between 3 and 5 years (Hughes, 1998; Carlson, 2005; Carlson et al., 2013), and continues to develop until early adolescence (Levin et al., 1991; Luna et al., 2004). Importantly, individual differences in WM have been shown to predict child outcomes in broad developmental domains, including language (Im-Bolter et al., 2006), mathematical skills (Bull and Scerif, 2001; Bull et al., 2008; Clark et al., 2010), and academic performance (Diamond et al., 2007).

Given the rapid development of and individual variability in WM during early childhood, researchers have hypothesized a relation between WM and children's transfer of learning from symbols (e.g., pictures, video) to real-world problems (Suddendorf, 2003; Barr, 2010; Troseth, 2010; Kirkorian, 2018). Unlike unmediated in-person experiences, symbol-mediated transfer requires children to obtain information from a context that involves a symbolic representation and then apply what they have learned to the symbol's referent in another context (Troseth and DeLoache, 1998; Troseth, 2010). Object-retrieval tasks are commonly used to study children's symbol-mediated learning. In such tasks, 2-year-olds who observe in-person hiding events outperform their peers who watch the same hiding events in a symbolic context, including scale models (DeLoache, 1987), photographs (DeLoache, 1991; DeLoache and Burns, 1994; Suddendorf, 2003), videos (Schmitt and Anderson, 2002; Sharon and DeLoache, 2003, Troseth and DeLoache, 1998; Troseth, 2003; Troseth et al., 2006, 2007; Schmidt et al., 2007; Kirkorian et al., 2016b), and touchscreens (Choi and Kirkorian, 2016; Kirkorian et al., 2016a).

Critically, while toddlers exhibit overall poorer performance in symbol-mediated object-retrieval tasks, detailed analyses of trial-by-trial performance sometimes reveal a more complex story. When learning from symbolic media, toddlers tend to show higher performance on the first trial than the following trials (Schmitt and Anderson, 2002; Sharon and DeLoache, 2003, Troseth and DeLoache, 1998; Schmidt et al., 2007). Most of the errors in the subsequent trials are due to perseveration, that is, searching in a previously correct location rather than the hiding location in the current trial (Troseth and DeLoache, 1998; Schmitt and Anderson, 2002). In other words, toddlers can use symbols to find hidden objects in the real world, but this may not be realized when the symbols conflict with the relatively salient real-life experience of finding the object on earlier trials. Researchers have proposed WM as a possible cognitive mechanism explaining perseverative errors in object-retrieval tasks (Suddendorf, 2003; Troseth, 2010; Kirkorian, 2018), given the cognitive resources required to update outdated representations or manage competition between multiple representations.

Studies that experimentally manipulated memory load provide evidence of the role of WM in children's transfer of learning from symbolic media (Suddendorf, 2003; Sheehan et al., 2020). For example, Suddendorf (2003) decreased WM updating demands by testing 24-month-olds once in each of four distinct rooms with different objects. This procedure was designed to remove the conflicts between representations in WM. To the same end, Sheehan et al. (2020) introduced a 24-h delay between trials, allowing time for 36-month-olds to forget irrelevant information such as hiding locations on prior trials. In both cases, the adjustments improved children's search performance compared to prior research, suggesting that children can use a symbol to guide their search if representational conflicts are resolved.

Further evidence for the relation between WM and symbol-mediated learning comes from studies on individual differences in cognitive control. Hartstein and Berthier (2018) tested children aged 33–39 months on WM, as well as inhibitory control and cognitive flexibility. They examined the relative contribution of these cognitive skills to children's performance in a symbolic retrieval task that required children to understand the link between a room and a scale model of the room. Individual differences in WM, but not inhibitory control or cognitive flexibility, predicted children's search performance. If the same mechanism is applied to children's transfer based on televised images, we would expect a similar pattern of findings in toddlers' transfer from video. Consistent with this hypothesis, we previously reported that toddlers' WM predicted transfer from non-interactive video during an object-retrieval task in a sample of children 27–34 months old (Choi et al., 2018).

### Working Memory and Contingent Video

Research findings on toddlers' learning from interactive media have been mixed (Sheehan and Uttal, 2016; Kirkorian et al., 2017; Kirkorian, 2018; Xie et al., 2018). On the one hand, several studies showed that toddlers may be more likely to transfer information from screen media to real-life contexts when they interact with the media in some way. For example, toddlers learned better from media interactions via video chat (Troseth et al., 2006; Nielsen et al., 2008; Roseberry et al., 2014; Myers et al., 2017), computer games (Lauricella et al., 2010), and touchscreen applications (Choi and Kirkorian, 2016; Kirkorian et al., 2016a; Huber et al., 2020) than from prerecorded video. On the other hand, some empirical evidence indicates that interactive learning does not always alleviate toddlers' transfer difficulties (Moser et al., 2015; Myers et al., 2018; Strouse et al., 2018; Troseth et al., 2018). Further, media interactivity sometimes hinders learning, as when young children played a digital game rather than watching recordings of someone else playing the game (Aladé et al., 2016; Schroeder and Kirkorian, 2016).

Differences in the nature of interactive media may partly explain mixed findings across studies. For instance, learning is likely to be affected by the extent to which reciprocal social interactions increase arousal and engagement (Kuhl, 2007), the extent to which interactivity places additional cognitive demands on the learner (Strommen, 1993; Fisch, 2016), and the extent to which interactive features draw children's attention toward vs. away from task-relevant information (Kirkorian, 2018). Some prior research integrates a broad range of interactive media features, including personal relevance (e.g., using the child's own name), reciprocity (e.g., responding differently depending on the child's own behavior; e.g., Troseth et al., 2006; Nielsen et al., 2008; Roseberry et al., 2014), and the ability to make choices that produce differential feedback (e.g., Schroeder and Kirkorian, 2016; Aladé et al., 2016). Thus, to constrain the ways in which media interactivity may impact learning, we focus on a narrower definition of *contingent video*, defined as linear video that plays in response to the child's physical action, as when tapping a touchscreen or computer key to play the next video (Lauricella et al., 2010; Choi and Kirkorian, 2016; Kirkorian et al., 2016a).

Even using a narrow definition of tap-to-play video contingency, mixed findings have been reported within studies. That is, the impact of contingency varied across children using the same simple touchscreen applications. In these studies, the effect of contingency appeared to vary as a function of child age (Choi and Kirkorian, 2016; Kirkorian et al., 2016a). Yet, while some existing research showed age-related changes in the impact of contingency on learning, less attention has been paid to the role of specific cognitive skills that may underlie age effects. Thus, despite emerging evidence that contingency can be both beneficial and detrimental for children's learning, the specific mechanisms that might lead to different outcomes remain unclear. Investigating individual differences in video-based transfer has the potential to reveal mechanisms behind the effect of contingent interactions with video. Such research would help to identify which children are affected by interactive media—for good or ill—and under what conditions, leading to evidence-informed guidelines for integrating interactive technology into education for young children (Valkenburg and Peter, 2013).

Scholars have proposed that WM may moderate the impact of contingent interactions on toddlers' learning from screens in multiple ways. For example, contingency may draw on greater cognitive resources than watching video because contingent video entails generating responses at the right place and time (Strommen, 1993; Fisch, 2016). Thus, interactions with screens may bring advantage only to children who already possess sufficient WM capacity to simultaneously watch and comprehend video content while operating a touchscreen device. In such a case, perhaps only toddlers with higher WM would learn better from contingent than non-contingent video, whereas there could be little or even negative effect for those with lower WM as the cognitive demands of contingency exceed available WM resources.

Alternatively, WM might moderate the impact of contingent video in the opposite direction. For instance, contingency might guide attention to essential information. In general, infants and young children allocate attention less systematically than adults, and they primarily deploy attention to perceptually salient features when watching video (Frank et al., 2009; Kirkorian et al., 2012; Franchak et al., 2016; Rider et al., 2018). Contingent interactions with a specific element on a screen may provide an attentional guide that reduces the number of items to be processed and, in consequence, lessen the cognitive load of processing video. Moreover, contingency may allow children to control the pace at which videos are presented. Such supports could be particularly useful for children who exhibit difficulty in holding and updating multiple pieces of information in memory. In this case, toddlers with lower WM would benefit from contingent interactions with video, whereas those with higher WM would transfer equally well from both contingent and non-contingent video. In other words, contingency would help to reduce gaps between children with lower vs. higher WM skills.

In summary, findings are mixed regarding the impact of contingency on toddlers' transfer from screen media. Differences in the type of interactive media cannot fully explain the mixed findings. Mixed findings appear within studies using relatively simple video contingency, with contingency effects sometimes varying by age within a single study. Thus, age-related cognitive skills such as WM may be able to predict which children are likely to learn from contingent vs. non-contingent video.

### Overview of the Current Study

The purpose of this study was to investigate the extent to which WM moderates the impact of contingency on toddlers' transfer from screen media to real-life stimuli. We used an object-retrieval task to observe learning across multiple trials. We compared toddlers (24–36 months) who viewed hiding events via contingent video that required children to touch a specific location on a screen to those who viewed hiding events on non-contingent video that advanced automatically without a touch response. After watching each hiding event, toddlers were asked to search for the hidden object on a corresponding felt board. In addition, toddlers' WM was assessed.

Based on prior research, we expected WM to predict object retrieval. Given mixed findings in prior research, the impact of contingency remained an open research question. Of particular interest was the extent to which WM moderated the effect of contingency on object retrieval, as evidenced by a WM-by-condition interaction. The nature of this interaction, if any, remained an open research question. Contingency could increase cognitive load (e.g., by requiring children to plan and execute responses to the screen) and therefore be most useful for children with relatively high WM capacity. Alternatively, contingency could decrease cognitive load (e.g., by guiding toddlers' attention or enabling them to control the pace of the video) and therefore be most useful for children with relatively low WM capacity.

## Methods

### Participants

Participants were 134 toddlers (48% females) between 24 and 36 months of age (mean_age_ = 30.0 months, SD_age_ = 2.9 months). Participants were assigned to one of two groups: contingency (*n* = 64, 42% females; mean_age_ = 30.0 months, SD_age_ = 2.8 months) or no-contingency [*n* = 70 including 22 from Choi et al. (2018), 53% females; mean_age_ = 29.9 months, SD_age_ = 3.0 months]. An additional 15 children (7 contingency, 8 no-contingency) were tested but excluded from analysis because of incomplete tasks (*n* = 5), absence on the 2nd day of testing (*n* = 5), or experimenter error (*n* = 5). Differences in the size of each group resulted from random assignment (rather than counterbalanced cells) at each study site. Participants were recruited from a small city and surrounding areas in the Upper Midwest, US. Both parental informed consent and child assent were obtained before participation.

We used a partial replication approach to create the pooled no-contingency comparison group in the current study (for similar approaches, see Barr et al., 2005, 2009; Brito et al., 2012). We previously reported that WM predicted search performance in a no-contingency sample of 22 children aged 27–34 months (Choi et al., 2018). In the current study, the same 22 children were pooled with an additional 48 children aged 24–36 months. All data were collected using the same stimuli and procedures. WM was lower in the newly recruited no-contingency sample (mean = 9.0, SD = 3.7) compared to the previously collected no-contingency sample (mean = 10.8, SD = 3.4), but the difference was not statistically significant, *t*(68) = −1.86, *p* = 0.067. Additionally, there was no difference in search performance between the newly recruited (mean = 2.0, SD = 1.1) and the previously collected no-contingency groups (mean = 2.1, SD = 1.3), *t*(64) = −0.30, *p* = 0.763. Consequently, the data were combined for the current analyses.

We conducted an a priori power analysis using *pwr.r.test* function from the pwr package (version 1.3-0; Champely, 2018). Based on the power analysis using pilot data, a sample size of ~145 subjects was required to detect an effect size of *f* ^2^ = 0.12 with α = 0.05 and power = 0.8 when 10 variables are included in a generalized linear mixed-effects model. After excluding 15 children because of attrition or experimenter error, a *post hoc* power analysis indicates that the final sample of 134 with an observed effect size of *f* ^2^ = 0.10 has a power of 0.7 to detect the effect of WM, including the WM by condition interaction.

### Materials

**Figure 1:**
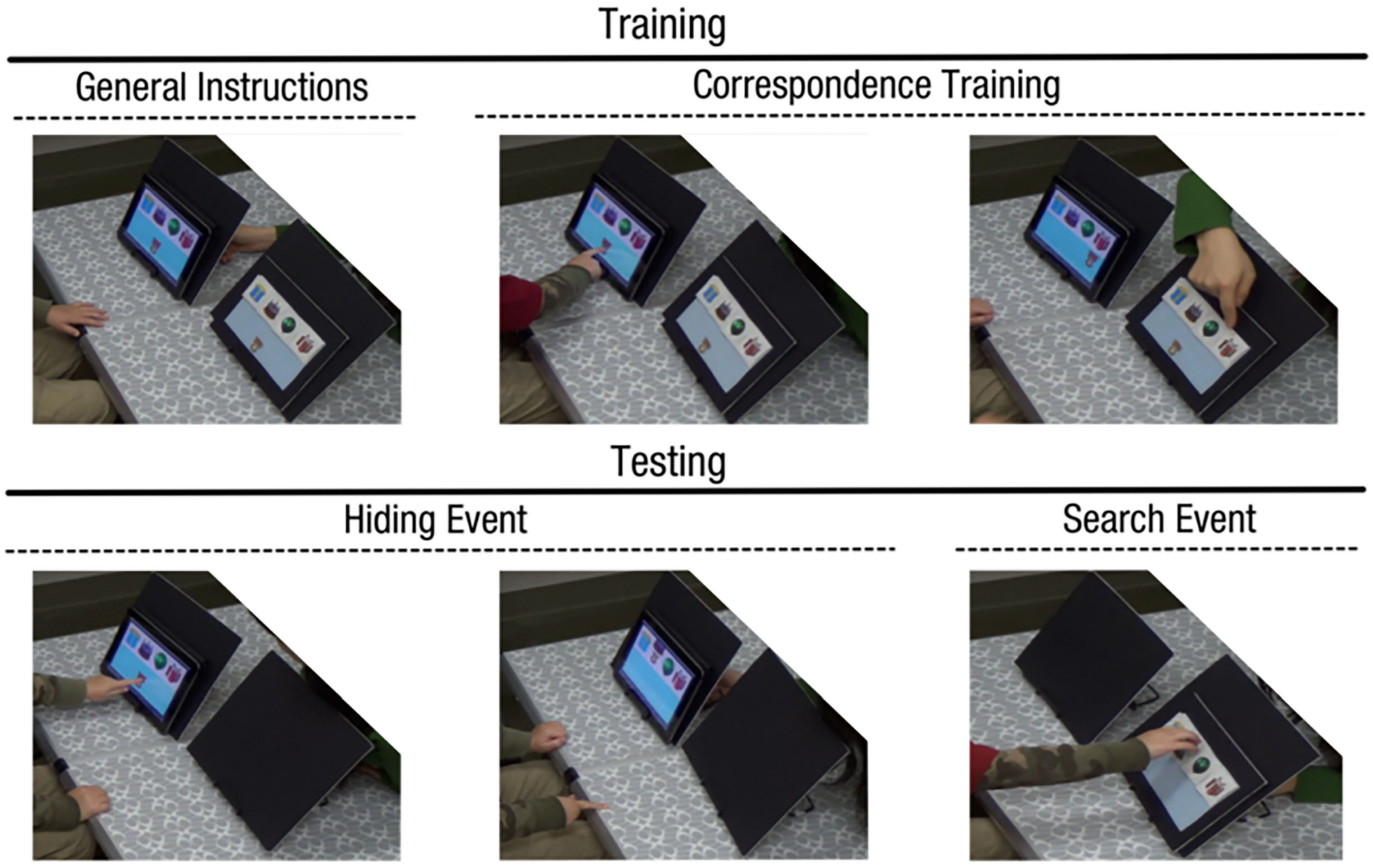
Object-retrieval task procedure for the contingency group.

The stimuli were the same as those used in our prior research (Choi and Kirkorian, 2016), which were adapted from earlier studies on toddlers' object retrieval from video (Schmidt et al., 2007; Kirkorian et al., 2016b) (Figure 1). The video stimuli used for hiding events showed a cartoon bear (2.5 × 2.5 cm) hiding behind four objects (4 × 4 cm each), which were animated to minimize the interactions with a live experimenter. The video stimuli were presented on a touchscreen tablet computer (10.1-inch Samsung Galaxy Tab) using a custom-built application. The only difference between the contingency and no-contingency groups was how the videos advanced during training and hiding events. For instance, in the no-contingency group, children watched the cartoon bear hide, and the video resumed automatically after a 1-s delay. In the contingency group, the child was instructed to touch the bear, and the image paused until the child touched the bear on the screen.

The felt board used for search events had the same size and appearance as the video stimuli. Images of the four objects were printed, cut, and loosely attached to the felt board so that children could lift them during their search. The bear image was printed on sticker paper so that children could manually retrieve it during their search.

### Procedure

Children were tested individually in an empty preschool classroom (43%) or a child-friendly laboratory room (57%). Preliminary analyses indicated that performance did not differ on WM or search performance for preschool vs. laboratory visits, *t*(132) = −1.49, *p* = 0.139, and *t*(128) = 0, *p* = 1.00, respectively. In preschools, children participated in two 20-min sessions spaced ~1 week apart. In the laboratory, children made one 45-min visit with a break after the first 20 min. Children sat alone or on the laps of their parents, directly across from an experimenter. An assistant noted children's responses and video-recorded children's behaviors. Children completed an object-retrieval task during the first preschool session (or the first half of the lab visit) and the WM assessment during the second preschool session (or second half of the lab visit).

### Object-Retrieval Task

In brief, the task consisted of three phases: (a) general instructions, (b) correspondence training, and (c) testing (Figure 1). During the general instructions phase, the experimenter first placed the tablet computer and the felt board on a table and introduced the task as a hide-and-seek game. During correspondence training, children watched the animated bear move below each of four hiding locations on the tablet. In the contingency group, children were instructed by the cartoon bear to first touch the bear before seeing it move to each location (e.g., “Touch me to see where I might hide”). In the no-contingency group, children were instructed by the cartoon bear to watch the bear hide. In both groups, the object wiggled as the cartoon bear said, “Sometimes I will hide here. Do you see this place on your friend's board?” Then the experimenter pointed to the corresponding locations on the felt board and said, “See this?” to highlight the correspondence between the tablet and felt board.

The correspondence training was followed by the testing phase involving four object-retrieval trials. Each testing trial consisted of a hiding event followed by a search event. During the hiding event, the felt board was occluded by a black board. Children watched the bear hide behind one of the four objects on the screen (only after touching the bear in the contingency group). During the search event, the experimenter occluded the tablet, hid the bear sticker on the felt board (outside of the child's view), and asked the child to find the sticker. In case of an incorrect search, children were encouraged to try another place until they found the sticker. Thus, each testing trial ended with retrieving the sticker. Children were congratulated for their correct search.

Children's performance during the search events was coded per trial. If a child searched the correct location on their first try, it was coded as an errorless search. Three children were missing the first trial data, and one child was missing the third trial data because of reluctance to search for the sticker. The experimenter demonstrated what to do, and all children searched on subsequent trials.

### WM

**Figure 2:**
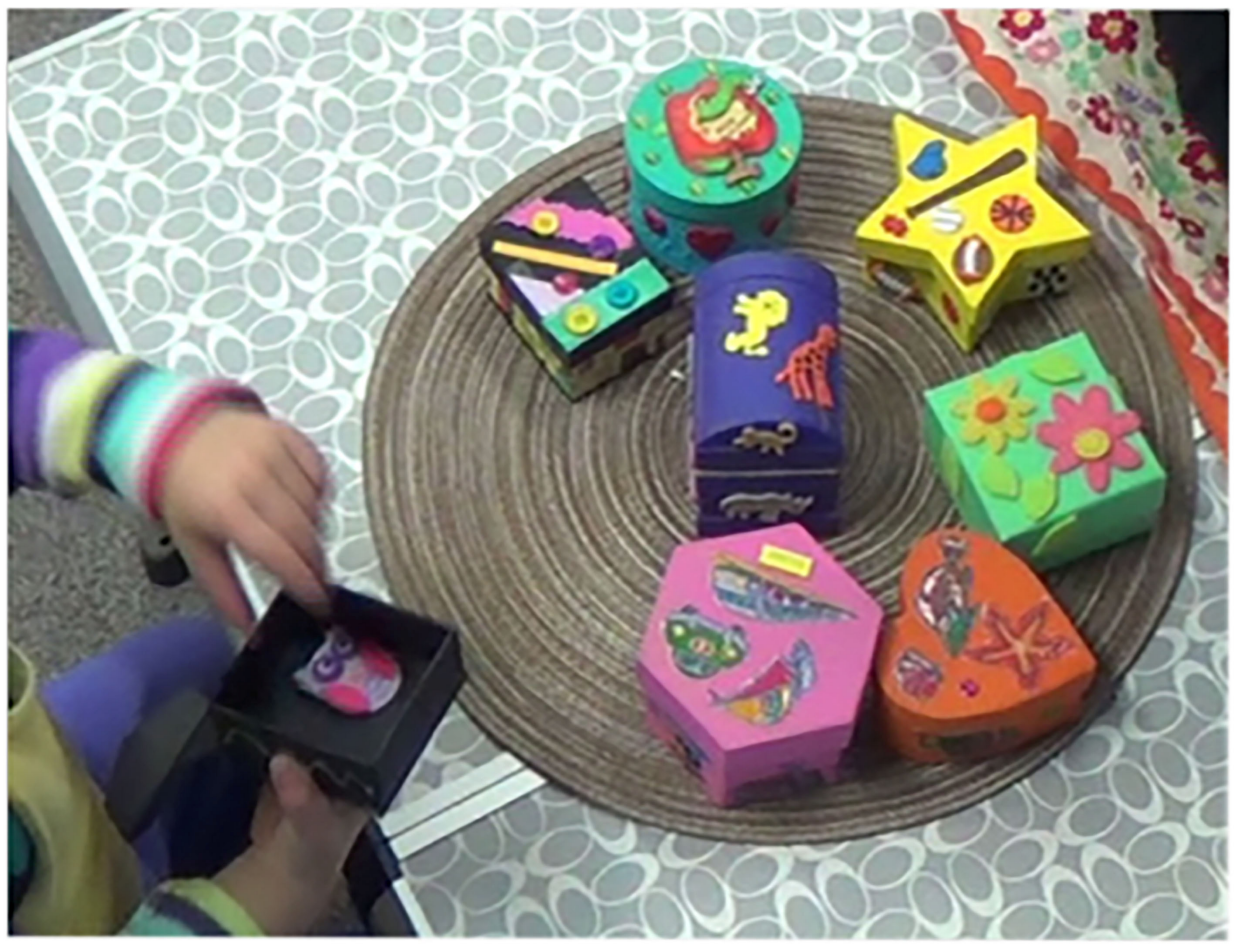
Spin the Pots task.

To assess WM, we adapted the Spin the Pots task from prior studies (Hughes and Ensor, 2005; Bernier et al., 2010; Brito et al., 2014) (Figure 2). The experimenter began by arranging a set of eight opaque boxes with distinct shapes and colors (e.g., decorated wooden boxes) on a round mat. For training, the experimenter hid one sticker inside a randomly chosen box and immediately asked the child to retrieve the sticker. This was repeated with one more box. For testing, the child watched the experimenter put a sticker in each of six different boxes, leaving two additional boxes empty. Subsequently, the experimenter covered the boxes with a cloth and rotated the mat 180 degrees. Then the experimenter removed the cloth and invited the child to find a sticker. The child was allowed to select only one box to open, after which the eight boxes were covered and rotated again. The task continued until all six stickers were found or the maximum number of 16 attempts was reached, whichever occurred first. Each child's WM score was given out of 16 after subtracting the number of errors (i.e., choosing a box in which no sticker had been hidden or in which the child searched on a previous trial). Possible scores ranged from 0 to 16, with higher scores indicating better WM. These scores were then standardized using *z* scores.

To estimate chance performance on the WM task, we simulated all possible outcomes given our task parameters (6 stickers, 8 boxes, 16 trials). This simulation resulted in a positively skewed distribution with a mode of 5 (29% of all possible outcomes) and nearly half of all possible outcomes (46%) producing a score ≤ 5. That is, if all children simply guessed at random, we would expect most scores to land near 5 with nearly half of children earning a score between 0 and 5.

### Parent Survey

Parents were asked to fill out an online survey about demographic information such as parent's education and child's race and ethnicity. Parents also reported whether they allow any touchscreen devices for their child and how much time (in minutes) their child spent doing each of the following activities on the previous day: (1) watching video content (TV, movies, portable video device), (2) using a computer (not Internet), (3) using the Internet, (4) using webcam or mobile device to video chat, (5) using a cellphone to talk to someone, (6) playing a video game console (e.g., Wii, PlayStation, Xbox), (7) playing a handheld video system (e.g., Nintendo DS. LeapFrog Leapster), (8) using a digital reading device (e.g., Nook, Kindle), and (9) using a touchscreen device, other than to call or video chat (e.g., iPad, touchscreen phone, Vinci, LeapPad). Daily screen time was operationalized as follows: (1) total usage reflects total minutes spent on any of screen media on the previous day; (2) TV/video usage represents minutes spent on viewing video on the previous day; (3) interactive media usage represents minutes spent on all interactive media (i.e., computer online, computer offline, video chat, voice chat, console game, handheld game, E-reader, touchscreen) on the previous day; and (4) touchscreen usage represents minutes spent using a touchscreen device, other than to call or video chat on the previous day.

### Analytical Approach

We first calculated descriptive statistics as well as bivariate and partial correlations to identify potential covariates. We also ran two-sample Kolmogorov–Smirnov tests to compare the distribution of observed WM scores to the distribution created by simulating all possible outcomes (i.e., the expected distribution based on random guessing). We then fit multilevel linear mixed-effects models to address our main research questions about the extent to which WM and contingency affect toddlers' object retrieval. Given that each trial had a binary outcome (errorless search = 1; error = 0), the model was specified with a binomial error structure and logit link function using the function *glmer* from the package lme4 (version 1.1-23; Bates et al., 2015) in the R software environment (version 4.0.2; R Core Team, 2020). Fixed effects included condition, age, and WM. Covariates included trial and gender. Participant was a random effect.

Prior research demonstrated that toddlers' object retrieval changes over trials (Schmitt and Anderson, 2002; Sharon and DeLoache, 2003, Troseth and DeLoache, 1998; Schmidt et al., 2007; Choi and Kirkorian, 2016; Kirkorian et al., 2016b). Specifically, children typically show an initial decrease in performance between the first and second trials due to perseveration, followed by a gradual increase in performance across subsequent trials. Because a non-linear trial effect was expected, we constructed a piecewise linear mixed-effects model with trials nested within participants. To specify two linear trajectories before and after Trial 2, the trial variable was recorded as two separate piecewise variables with a knot fixed at Trial 2, labeled as Phase 1 and Phase 2. Phase 1 was coded to indicate the change in children's search performance across the first two trials (Trial 1 = −1, Trial 2 = 0 Trial 3 = 0, Trial 4 = 0). Phase 2 was coded to index the change over the remaining trials (Trial 1 = 0, Trial 2 = 0, Trial 3 = 1, Trial 4 = 2).

The rest of the fixed effects were between-subject predictors. Condition was represented by a dummy variable, comparing the coded group (no-contingency) to the reference group (contingency). Child gender was dummy coded (reference category: male, coded category: female). Continuous predictors included age and WM, each standardized using *z* scores and centered at their minimums.

Our primary research question involved the extent to which WM moderated the impact of contingency on toddlers' object retrieval, controlling for age. However, an initial visual inspection of the data suggested that the effects of WM and contingency were themselves moderated by age. To confirm the interactive effects of condition, age, and WM, we performed a log-likelihood ratio (LLR) test. A full model with all interaction terms, including the three-way interaction, was compared with a reduced model without the three-way interaction term. Based on the LLR test, the full model with all interaction terms, including the three-way condition × age × WM interaction, provided a better fit for the data relative to the reduced model without the three-way interaction term, χ^2^(1) = 6.06, *p* = 0.014.

To estimate effect sizes for the multilevel linear mixed-effects models, we calculated semipartial *R*^2^ for each fixed effect to describe the proportion of variance explained by each fixed effect using the *r2beta* function from the r2glmm package (version 0.1.2; Jaeger, 2017). According to Cohen's (1992) guideline, semipartial *R*^2^ ≥ 0.02 can be interpreted as small effects, ≥0.13 as medium effects, and ≥0.26 as large effects (Page-Gould, 2017).

## Results

### Preliminary Analyses

#### Demographic and Media Use Characteristics

Table 1 presents descriptive statistics for participant demographic and media use characteristics for the 96 parents (72%) who returned the survey. Data are presented separately for the full sample (*N* = 134), the contingency group (*n* = 62), and the no-contingency group (*n* = 70). However, there were no systematic differences between the contingency and no-contingency groups for any of these characteristics. Thus, the description of descriptive statistics below is based on the full sample.

**Table 1:** Descriptive statistics for participant demographic and media use characteristics.

**Characteristics**	**Full sample** **(*n* = 134)**	**Contingency** **(*n* = 64)**	**No contingency** **(*n* = 70)**
	**Mean (SD)** **or n (%)**	**Mean (SD)** **or n (%)**	**Mean (SD)** **or n (%)**
Parent			
Education (years)	18.8 (3.2)	19.5 (3.2)	18.2 (3.1)
Missing	40 (29.9%)	19 (29.7%)	21 (30.0%)
Child			
Age (months)	30.0 (2.9)	30.0 (2.8)	29.9 (3.0)
Gender (female)	64 (47.8%)	27 (42%)	37 (53%)
Race and/or ethnicity			
White non-hispanic	72 (53.7%)	38 (59.4%)	34 (48.6%)
Other	23 (17.2%)	10 (15.6%)	13 (18.5%)
Missing	39 (29.1%)	16 (25.0%)	23 (32.9%)
Touchscreen rule (allowed)	76 (56.7%)	36 (56.2%)	40 (57.1%)
Missing	38 (28.4)	16 (25.0%)	22 (31.4%)
Daily screen time (min)			
Total usage (min)	37.5 (38.5)	35.7 (38.9)	39.2 (38.5)
Missing	30 (22.4%)	14 (21.9%)	16 (22.9%)
TV/video usage (min)	29.0 (30.7)	25.5 (31.0)	32.5 (30.2)
Missing	39 (29.1%)	17 (26.6%)	22 (31.4%)
Interactive media usage (min)	12.6 (23.6)	11.9 (24.2)	13.2 (23.3)
Missing	36 (26.9%)	15 (23.4%)	21 (30.0%)
Touchscreen usage (min)	9.0 (19.6)	6.63 (16.1)	11.4 (22.5)
Missing	36 (26.9%)	15 (23.4%)	21 (30.0%)

Parents' average years of education was roughly equivalent to a master's degree (mean = 18.8 years, SD = 3.2 years, range = 12–28 years). Most parents (76%) identified their child as white/Caucasian and non-Hispanic. A majority of parents (79%) allowed their children to use a touchscreen device at home. Children's total daily screen time reported by parents averaged 37.5 min (SD = 38.5 min, range = 0–165 min). Consistent with nationally representative surveys in the United States (Rideout, 2017), non-interactive TV and video viewing remained dominant (mean = 29.0, SD = 30.7, range = 0–120) compared to all interactive media usage (mean = 12.6, SD = 23.6, range = 0–130). Focusing specifically on touchscreen device use, parents reported 9 min on average (SD = 19.6 min, range = 0–120 min).

#### Correlations

**Figure 3:**
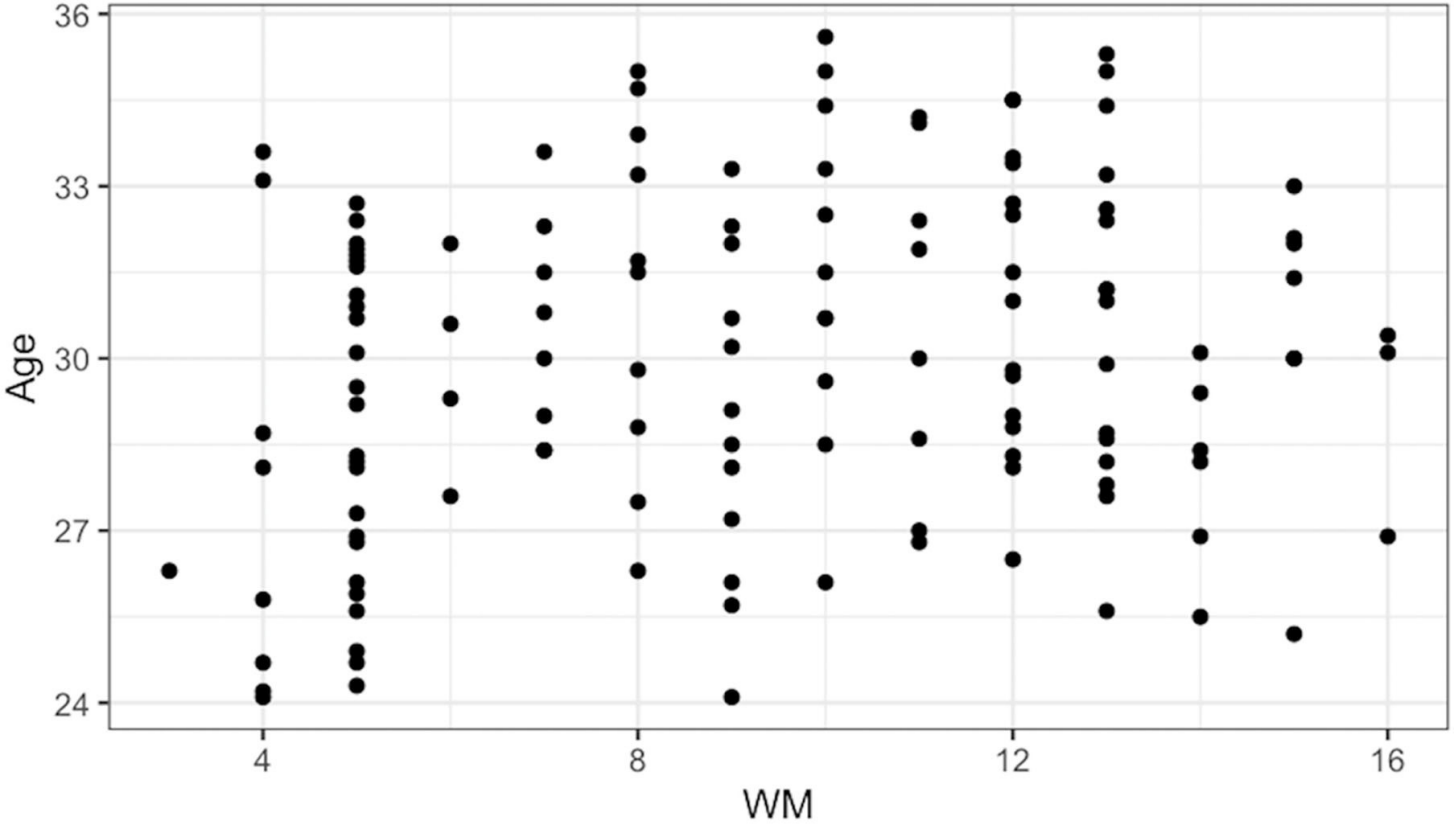
Scatterplot of age (in months) and WM scores.

Correlations between demographics, media use, WM, and object retrieval performance are presented in Table 2. Child gender was significantly correlated with WM and search performance*, r*(132) = 0.25, *p* = 0.003, and *r*(128) = 0.22, *p* = 0.013, respectively. Therefore, child gender was included as a covariate in subsequent analyses. Age was correlated with WM, *r*(132) = 0.20, *p* = 0.023 (Figure 3). The distribution of WM scores suggests a cluster of children surrounding a score of 5, as expected by our simulation of all possible outcomes. Nonetheless, the distribution of observed WM scores differed from the distribution of all possible outcomes (*D* = 0.47, *p* < 0.001), even when considering only children younger than 28 months (*D* = 0.35, *p* < 0.001). Moreover, the direction of effects reported later in this article remained the same even after excluding the 34 children (25%) who earned a score of 5 or less on the WM task. Of particular relevance to our current aims, age and WM were also correlated with search performance, *r*(128) = 0.43, *p* < 0.001, and *r*(128) = 0.25, *p* = 0.005, respectively. The rest of the child and family variables were not correlated with variables of interest and thus not considered further.

**Table 2:** Zero-order bivariate correlations between assessments (below diagonal) and partial correlations controlling for age (above diagonal).

	**1**.	**2**.	**3**.	**4**.	**5**.	**6**.	**7**.	**8**.	**9**.
1. Child age (months)	–	–	–	–	–	–	–	–	–
2. Child gender	0.04	–	0.09	0.19	0.15	0.02	0.13	0.25^**^	0.22^*^
3. Parent education (years)	0.08	0.10	–	−0.04	−0.09	0.11	0.18	0.19	−0.09
4. Total screen time (min)	0.02	0.19	−0.03	–	0.80^***^	0.60^***^	0.46^***^	0.16	0.08
5. TV/Video (min)	0.03	0.16	−0.09	0.80^***^	–	0.02	0.01	0.10	0.01
6. Interactive media time(min)	0.07	0.02	0.11	0.60^***^	0.02	–	0.08^***^	0.09	0.08
7. Touchscreen time (min)	0.12	0.14	0.19	0.46^***^	0.01	0.80^***^	–	0.10	0.06
8. WM	0.20^*^	0.25^**^	0.20	0.17	0.10	0.10	0.12	–	0.19^*^
9. Search performance	0.43^***^	0.22^*^	−0.03	0.08	0.02	0.10	0.11	0.25^**^	–

Variables included child age (months), gender (0 = male, 1 = female), parent education (years), total screen time at home (minutes yesterday), TV/video time at home (minutes yesterday), interactive screen time at home (minutes yesterday), touchscreen time at home (minutes yesterday), the working memory score, and the total correct searches across all trials. The correlation matrix included Pearson correlations for continuous variable pairs (e.g., child age–parent education) and point-biserial correlations for continuous-dichotomous pairs (e.g., child age–gender).

* p < 0.05,

** p < 0.01,

*** *p < 0.001*.

### Probability of Errorless Search as a Function of Condition, Age, and WM

**Figure 4:**
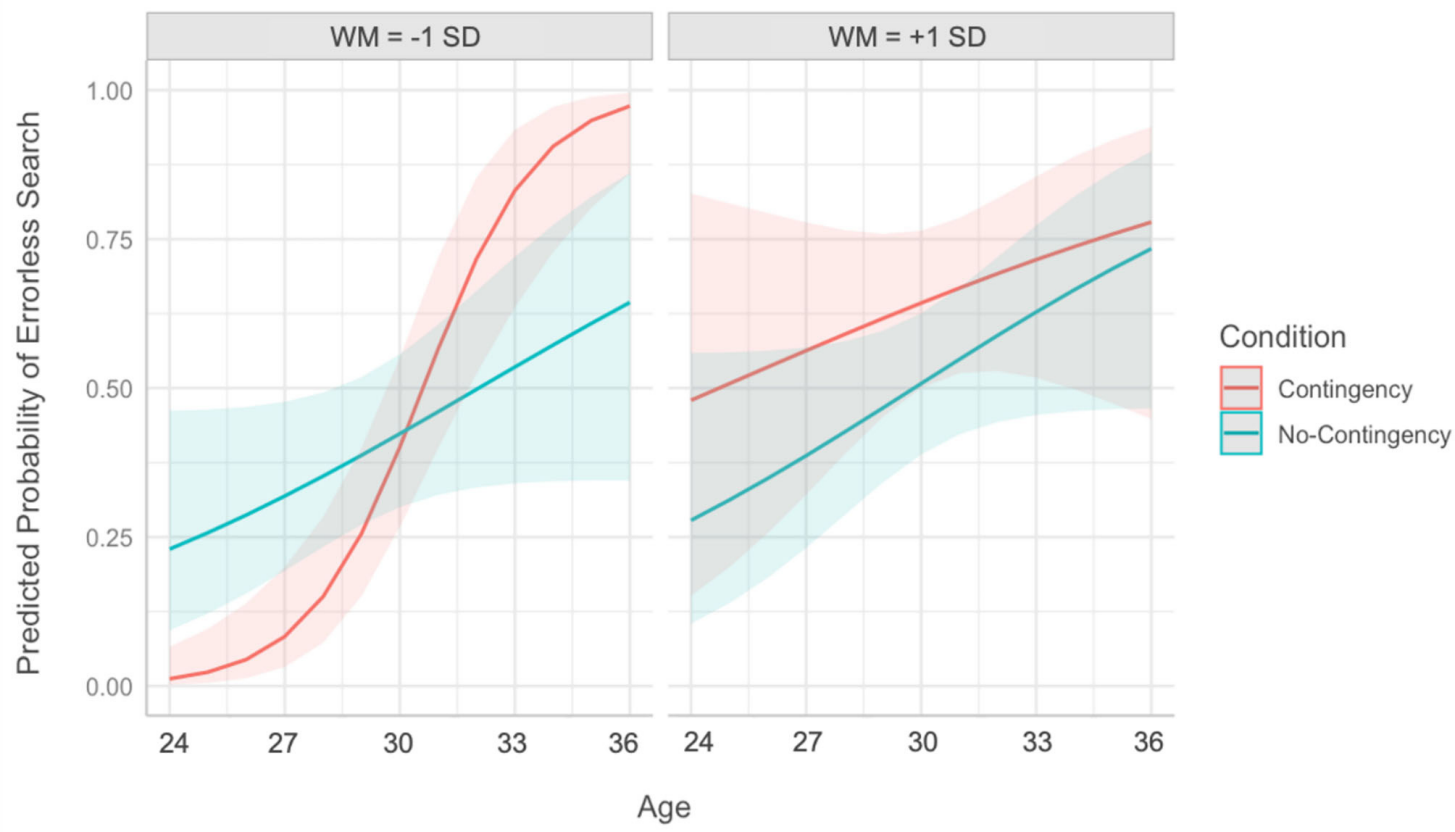
Predicted probability of errorless search as a function of age and condition plotted at −1 SD **(left)** and +1 SD from the mean WM **(right)**, controlling for gender and trial effects. WM was a continuous predictor in the model. The shaded bands represent the 95% confidence intervals.

Overall, 49.6% of trials were errorless in the no-contingency group, and 50.4% of trials were errorless in the contingency group. Table 3 presents fixed and random effects for the full model predicting the probability of an errorless search as a function of condition, age, and WM, controlling for trial (i.e., Phase 1 and Phase 2) and gender. The model results are also plotted in Figure 4 to illustrate the impact of contingency on object retrieval among children with varying age and WM.

**Table 3:** Fixed effects from the final mixed logit model predicting the probability of errorless search.

**Predictors**	**β (SE)**	***z***	**OR**	**95% CI (OR)**
(Intercept)	−7.23 (1.35)	−5.38^***^	0.00	[0.00, 0.01]
Age (centered at 24 months)	2.56 (0.60)	4.23^***^	12.89	[3.94, 42.13]
WM (centered at the lowest score)	2.09 (0.64)	3.28^**^	8.12	[2.32, 28.42]
Condition (no contingency)	4.52 (1.46)	3.09^**^	92.07	[5.23, 1621.54]
Age × WM	−0.82 (0.29)	−2.79^**^	0.44	[0.25, 0.78]
Condition × age	−2.07 (0.69)	−3.00^**^	0.13	[0.03, 0.49]
Condition × WM	−1.99 (0.73)	−2.71^**^	0.14	[0.03, 0.58]
Condition × age × WM	0.82 (0.34)	2.40^*^	2.26	[1.16, 4.40]
Phase 1 (change across trials 1–2)	−2.21 (0.32)	−6.82^***^	0.11	[0.06, 0.21]
Phase 2 (change across trials 2–4)	0.78 (0.16)	4.95^***^	2.17	[1.60, 2.95]
Gender (female)	0.57 (0.28)	2.05^*^	1.77	[1.03, 3.06]
**Random effects**				
σ^2^	3.29			
τ_00__ID_	0.84			
ICC	0.20			
N_ID_	134			
Observations	532			
Marginal *R*^2^/conditional *R*^2^	0.343/0.477			

Predictors were child gender (0 = male, 1 = female), Phase 1 (testing change across trials 1–2), Phase 2 (testing change across trials 2–4), age (standardized using z scores and centered at 24 months), WM (standardized using z scores and centered at the lowest score), and condition (0 = contingency, 1 = no contingency). OR, odds ratio; CI, confidence interval.

* p < 0.05,

** p < 0.01,

*** *p < 0.001*.

Both age and WM were centered at their minimums in this model. Therefore, the fixed effects presented in Table 3 are based on a reference group of the youngest children with the lowest WM. In this model, there was a significant effect of condition such that search performance was higher in the no-contingency (coded) group than the contingency (reference) group, β = 4.52, SE = 1.46, odds ratio (OR) = 92.07, *p* = 0.002, $R_{\beta }^{2}$ = 0.04. That is, among the youngest children with the lowest WM, object retrieval was higher for no-contingency than contingency. Moreover, in the contingency (reference) group, search performance generally increased with age and WM, β = 2.56, SE = 0.60, OR = 12.89, *p* < 0.001, $R_{\beta }^{2}$ = 0.08; and β = 2.09, SE = 0.64, OR = 8.12, *p* = 0.001, $R_{\beta }^{2}$ = 0.05, respectively.

Significant interactions indicated that the impact of contingency varied across both age and WM. To identify the children for whom contingency had a significant effect, we conducted analogous *post hoc* models, recentering age and WM to change the reference group (not shown in Table 3). First, we recentered age at its maximum instead of its minimum. This *post hoc* model confirmed that the condition effect reversed among the oldest toddlers in the sample, with a higher rate of errorless search in the contingency group than the no-contingency group for the oldest toddlers with the lowest WM score, β = −3.72, SE = 1.53, OR = 0.02, *p* = 0.015, $R_{\beta }^{2}$ = 0.02. That is, contingency increased object retrieval among children with the lowest WM when considering those at the oldest end of the age range. Next, we recentered WM at its maximum to examine the effect of contingency among toddlers with the highest WM. The condition effect was not significant among these higher WM children, whether age was centered at its minimum or maximum, β = −2.73, SE = 1.53, OR = 0.07, *p* = 0.075, $R_{\beta }^{2}$ = 0.01; and β = 0.84, SE = 1.41, OR = 2.32, *p* = 0.551, $R_{\beta }^{2}$ = 0.001, respectively. Thus, contingency did not affect search performance for children with the highest WM, regardless of age.

The full model in Table 3 also revealed changes in object-retrieval performance across trials, including a significant linear decrease between Trials 1 and 2 (Phase 1) and a significant linear increase between Trials 2 and 4 (Phase 2), β = −2.21, SE = 0.32, OR = 0.11, *p* < 0.001, $R_{\beta }^{2}$ =0.09; and β = 0.78, SE = 0.16, OR = 2.17, *p* < 0.001, $R_{\beta }^{2}$ = 0.04, respectively. Together, these effects reflected a V-shaped pattern such that performance initially decreased sharply between the first and second trials and then increased gradually over the subsequent trials. The gender effect was also significant, such that girls showed higher performance than boys, β = 0.57, SE = 0.28, OR = 1.77, *p* = 0.040, $R_{\beta }^{2}$ = 0.01. The V-shaped pattern of trial effects and the gender effect remained significant in the rotated models (not shown in Table 3).

## Discussion

Previous research reveals mixed findings for the impact of interactivity on toddlers' screen-based transfer (Lauricella et al., 2010; Choi and Kirkorian, 2016; Kirkorian et al., 2016a; Russo-Johnson et al., 2017; Huber et al., 2020). Scholars have proposed that WM may moderate toddlers' learning in different ways, such as increasing cognitive load (Strommen, 1993; Fisch, 2016) or guiding visual attention to important information (Kirkorian, 2018). The purpose of this study was to investigate the extent to which individual differences in WM moderated the impact of a simple tap-to-play contingency on screen-mediated object retrieval.

We first consider a scenario in which contingency creates additional cognitive load, supporting transfer among only those toddlers with sufficient WM capacity and potentially hindering transfer among those with lower WM. This scenario was partially supported in the current study. Specifically, this scenario is consistent with our findings for the youngest toddlers in our sample. When our model was centered on children 24 months of age, contingency decreased search performance among toddlers with relatively low WM. On the other hand, contingency did not hinder performance when the youngest children had relatively high WM. Indeed, there was a (non-significant) trend for contingency, relative to no-contingency, to increase rather than decrease search performance among the youngest children with the highest WM. Thus, contingency may have a different effect for younger toddlers with adequate WM capacity.

Another scenario we considered was that contingency guides children's attention and reduces the amount of information to be processed, making it particularly useful for children with relatively low WM. This scenario could potentially explain the pattern of results among the oldest toddlers in our study. When our model was centered on children 36 months of age, contingency increased search performance among those with relatively low WM, whereas older toddlers with relatively high WM did well, regardless of condition. In other words, the facilitative effect of contingent video was most evident for the oldest toddlers who were less able to maintain and update multiple representations. Perhaps in these older toddlers, low WM can be overcome by working in conjunction with another age-related skill not measured in this study, such as representational insight (Troseth, 2010), representational flexibility (Barr, 2010), attention shifting (Garon et al., 2008), or inhibitory control (Russo-Johnson et al., 2017). This coordination of skills may enable older children to take advantage of contingency in ways not accessible to their younger counterparts.

Together, our results reveal a moderating role of WM in the impact of contingency on children's screen-based object retrieval. Prior research showed that WM accounted for variability in young children's (33–39 months) transfer between a symbol (i.e., scale model room) and referent (i.e., real life-sized room; Hartstein and Berthier, 2018). Similarly, we previously reported that WM predicted young children's (27–34 months) performance in a search task using non-contingent video (Choi et al., 2018). In the current study, we extend this finding to contingency in interactive screen media, elucidating who is most likely to be affected by contingent interactions with the screen. Our findings add evidence that transfer from symbols—including contingent video—is cognitively taxing and thus requires WM resources, at least for young toddlers (Schmidt et al., 2007; Barr, 2010; Troseth, 2010). Therefore, a child's ability to maintain and update relevant information in memory appears to be important in the successful transfer of symbol-mediated learning that involves contingent interactions.

Here we found the contingency effect on children's object retrieval was modified not only by WM but also by age. Earlier studies have been mixed regarding the impact of contingency on toddlers' learning from screen media. The effects of media contingency (either touching the screen or pressing a computer key) were greatest among the youngest toddlers in some studies (Choi and Kirkorian, 2016; Kirkorian et al., 2016a) but not in others (Lauricella et al., 2010; Russo-Johnson et al., 2017; Huber et al., 2020). Sample differences in WM could explain why the age effect observed in the current study appears to contradict earlier studies in which contingency was more likely to benefit younger toddlers than older toddlers (Choi and Kirkorian, 2016; Kirkorian et al., 2016a). Further, the extent to which contingency is beneficial may depend on the relative difficulty of the lesson (Kirkorian, 2018). For example, tasks that involve multiple trials with several distractors may require memory updating, resulting in higher cognitive load than other tasks (Barr, 2010). Indeed, Strouse and Samson (2020) revealed in their meta-analysis that object retrieval studies showed more difficulties in transfer than other learning domains such as word learning and imitation. Thus, there may be subtle differences in task complexity included in the current study that resulted in a different “peak” for the contingency effect either directly or interacting with other factors such as age.

### Limitations and Future Directions

This study has limitations that must be addressed by future studies to fully describe associations between WM, contingency, and transfer using symbolic media. First, the current study leverages multiple data sets. For practical reasons, we used a synergistic approach, pooling data from different rounds of data collection to address the unique research questions described here. We did not find evidence of systematic variability due to cohort effects or differences in study protocols. Nonetheless, all of these small variations could add up to create differences in children's responses in subtle ways that were not detectable in the current study. We also note that our study was underpowered according to the *post hoc* power analysis. Therefore, the findings should be interpreted with caution and replicated in future work to confirm that the findings are robust beyond the samples used here.

Relatedly, this study relies on a relatively homogenous convenience sample. Although parent's education was not associated with WM or search performance, there was relatively little variability in this highly educated sample, perhaps masking effects that might be found in samples with more variability in parent education. Given the positive relation between socioeconomic status and WM (e.g., Hackman et al., 2014), it is also possible that this homogeneous sample may reduce potential variation in children's WM performance, limiting the generalizability of the study results. Subsequent studies should seek to replicate the results using a larger and more diverse sample.

Other limitations exist in our measurement of WM. We used a single WM task assessing an age range that involves substantial cognitive development (Carlson, 2005; Garon et al., 2008). Our scoring scheme (16—the total number of errors made) is limited in distinguishing whether errors occurred earlier or later in the trials. That is, children who found five of the six stickers right away could receive the same WM scores as another who retrieved five stickers non-consecutively across 16 trials. In this way, scores on the WM task may be capturing variability in abilities other than WM. Nonetheless, it does seem that this task is capturing meaningful variability above and beyond age. Most notably, we do get the same pattern of results with regard to condition effects and interactions when examining only the first eight WM trials or when excluding children with relatively low scores that may indicate random guessing. We decided to use the full 16 trials to be consistent with the scoring scheme used in prior work (Hughes and Ensor, 2005; Bernier et al., 2010; Brito et al., 2014) and to maximize the variability that is captured by the task. However, future work should utilize multiple converging, age-appropriate measures to isolate the effects of WM vs. other cognitive abilities in young children's learning from contingent interactions with symbolic media.

Another important future direction will be establishing the generalizability of the findings across different types of interactive screen media. Our manipulation involved a simple video contingency in which children tapped the screen to play each video. WM appears to moderate the effects of even this simple contingency. The extent to which WM moderates other types of interactivity, such as open-ended game play or reciprocal video chat, remains to be seen and likely depends on the extent to which such media create vs. alleviate cognitive burden (Strommen, 1993; Fisch, 2016; Kirkorian, 2018).

Finally, future work should consider other factors that may elucidate individual differences in the optimal conditions for learning. For instance, consistent with prior studies (Kotsopoulos et al., 2017; Zimmermann et al., 2021), we found that girls performed better than boys on our version of the Spin the Pots task. As our primary goal in this study was to examine the moderating role of WM, we decided to include gender as a covariate in our models to control for its effect rather than examining it as a moderator. Gender differences have been reported in other studies on young children's learning from touchscreens (e.g., Russo-Johnson et al., 2017) and may be worthy of future study.

Similarly, while WM may explain why the age effect in the current study appears inconsistent with previous research on this topic, there are alternative explanations. The differences found in this study may be explained by other age-related skills, such as inhibitory control, which are not accounted for in the current study (Russo-Johnson et al., 2017). Thus, future research should examine the role of this and other possible mechanisms (e.g., other cognitive characteristics, task differences) to fully capture the individual differences that may moderate media effects.

## Conclusion

Together, our findings suggest that WM is one—but not the only—moderator of young children's screen-based transfer. These findings corroborate the importance of considering different child characteristics in understanding media effects on children (Fisch, 2000, 2016; Valkenburg and Peter, 2013). Moreover, WM may help to explain mixed findings in prior research. However, our finding that age contributes to explaining the interaction between contingency and WM suggests the possibility of other age-related skills not examined here. Our findings emphasize the coaction among child and media characteristics in influencing children's learning from symbolic media. Identifying which children are most likely to benefit from contingent vs. more receptive, observational experiences would be an important step toward understanding how individual children learn from various symbolic media. Individual differences in learning from different symbolic media could compound over time, setting children on a trajectory with implications for longer-term developmental outcomes.

## Data Availability Statement

The raw data supporting the conclusions of this article will be made available by the authors, without undue reservation.

## Ethics Statement

The studies involving human participants were reviewed and approved by the University of Wisconsin-Madison. Written informed consent to participate in this study was provided by the participants' legal guardian/next of kin.

## Author Contributions

KC conceptualized the study in collaboration with HK and TP. HK and TP secured funding. KC and HK designed the study and collected data. KC analyzed the data and wrote the first draft of the manuscript. HK provided substantial feedback on data analysis and writing. TP provided feedback on manuscript drafts. All authors approved the submitted version of the manuscript.

## Conflict of Interest

The authors declare that the research was conducted in the absence of any commercial or financial relationships that could be construed as a potential conflict of interest.
